# Identifying Breast Cancer Recurrence in Administrative Data: Algorithm Development and Validation

**DOI:** 10.3390/curroncol29080424

**Published:** 2022-07-28

**Authors:** Claire M. B. Holloway, Omid Shabestari, Maria Eberg, Katharina Forster, Paula Murray, Bo Green, Ali Vahit Esensoy, Andrea Eisen, Jonathan Sussman

**Affiliations:** 1 Disease Pathway Management, Clinical Institutes and Quality Programs, Ontario Health, 525 University Avenue, Toronto, ON M5G 2L3, Canada; katharina.forster@ontariohealth.ca; 2 Department of Surgery, University of Toronto, 149 College Street, Toronto, ON M5T 1P5, Canada; 3 Institute of Health Policy, Management, and Evaluation, University of Toronto, 155 College Street 4th Floor, Toronto, ON M5T 3M6, Canada; omid.shabestari@utoronto.ca (O.S.); ali.esensoy@utoronto.ca (A.V.E.); 4 Data and Decision Sciences, Health System Performance and Support, Ontario Health, 525 University Avenue, Toronto, ON M5G 2L3, Canada; maria.eberg@mail.mcgill.ca (M.E.); paula.marguerite7@gmail.com (P.M.); 5 Quality Measurement and Evaluation, Clinical Institutes and Quality Programs, Ontario Health, 525 University Avenue, Toronto, ON M5G 2L3, Canada; bo.green@ontariohealth.ca; 6 Medical Oncology, Sunnybrook Health Sciences Centre, 2075 Bayview Avenue, Toronto, ON M4N 3M5, Canada; andrea.eisen@sunnybrook.ca; 7 Department of Oncology, McMaster University, 699 Concession Street Suite 4-204, Hamilton, ON L8V 5C2, Canada; sussman@hhsc.ca

**Keywords:** breast neoplasms, neoplasm recurrence, local, recurrence, algorithms, outcome assessment, healthcare, predictive value of tests, diagnostic techniques and procedures, prevalence, humans, cohort studies

## Abstract

Breast cancer recurrence is an important outcome for patients and healthcare systems, but it is not routinely reported in cancer registries. We developed an algorithm to identify patients who experienced recurrence or a second case of primary breast cancer (combined as a “second breast cancer event”) using administrative data from the population of Ontario, Canada. A retrospective cohort study design was used including patients diagnosed with stage 0-III breast cancer in the Ontario Cancer Registry between 1 January 2009 and 31 December 2012 and alive six months post-diagnosis. We applied the algorithm to healthcare utilization data from six months post-diagnosis until death or 31 December 2013, whichever came first. We validated the algorithm’s diagnostic accuracy against a manual patient record review (*n* = 2245 patients). The algorithm had a sensitivity of 85%, a specificity of 94%, a positive predictive value of 67%, a negative predictive value of 98%, an accuracy of 93%, a kappa value of 71%, and a prevalence-adjusted bias-adjusted kappa value of 85%. The second breast cancer event rate was 16.5% according to the algorithm and 13.0% according to manual review. Our algorithm’s performance was comparable to previously published algorithms and is sufficient for healthcare system monitoring. Administrative data from a population can, therefore, be interpreted using new methods to identify new outcome measures.

## 1. Introduction

Breast cancer recurrence is an important outcome for patients and healthcare systems, but recurrence is not routinely reported in cancer registries or other administrative datasets [1,2,3,4]. Ontario Health (Cancer Care Ontario) is an agency of the government of Ontario, Canada, that measures cancer system performance, among other functions. Measuring breast cancer recurrence in the population of Ontario could inform healthcare system planning and quality improvement since recurrence has been associated with modifiable factors such as margin positivity after surgery [5,6] and treatment selection [5,7,8], and treating recurrence requires significant healthcare resources [9]. Moreover, many breast cancer survivors worry about recurrence [10,11] and both recurrences and second primary breast cancers have been associated with reduced survival [5,12,13], so recurrence rates could inform discussions of risk.

The gold standard for identifying cancer recurrence is a manual review of patient information, which is not feasible at the population level. Researchers have used other methods to identify breast cancer recurrences, such as surveying patients directly [14], or developing algorithms for identifying breast cancer recurrences [3,15,16,17,18] or second breast cancer events (SBCEs) [1,2,19], which combine local and distant recurrences and second primary breast cancers. However, at the population level, patient surveys are impractical, and some algorithms may not be appropriate: some algorithms have been developed from highly selected breast cancer cohorts (potentially with specific treatment patterns), and some did not identify second primary breast cancers as well as local and distant recurrences. Developing an algorithm that could be applied across a population could support system-level decision making, increase algorithm generalizability, and ensure sufficient numbers of SBCEs to provide precise estimates of algorithm accuracy since breast cancer recurrence rates are generally low. Since algorithms developed in other jurisdictions would need to be validated before they could be applied to the Ontario population, and some existing algorithms incorporate data that are inaccessible in Ontario or Canada, we aimed to:

(1) Develop a novel algorithm for measuring SBCE rates (recurrences and second primary breast cancers) in a population using routinely collected administrative data;
(2) Validate the algorithm’s diagnostic accuracy using the results of a manual record review in a large sub-cohort of patients.

For this study, we defined an SBCE as evidence of a local, regional, or distant breast cancer recurrence or a new primary breast cancer observed more than 180 days after the incident breast cancer diagnosis.

## 2. Materials and Methods

### 2.1. Patient Selection and Data Sources

This retrospective cohort study included all female patients 18 years old or older diagnosed with stage 0-III breast cancer in the Ontario Cancer Registry [20] between 1 January 2009 and 31 December 2012. Patients with a prior diagnosis of breast or other cancer were included, as prior diagnoses were not expected to change the outcome of interest (detection of recurrence after the incident date). Healthcare utilization data from incident diagnosis until 31 December 2013 or patient death, whichever came first, were retrieved for analysis. Patients were excluded if they were diagnosed with lymphoma in the breast or skin cancer on the breast or died within 180 days (six months) of diagnosis.

Patients’ unique Ontario Health Insurance Plan numbers [21] were used to link data. The Ontario Registrar General provided the cause-of-death data. Stage data, including tumor characteristics, were retrieved from the Ontario Cancer Registry [20]. Inpatient procedure data, including associated diagnosis codes, were retrieved from the Discharge Abstract Database [22]. Emergency department visit data, outpatient procedure data, and associated diagnosis codes were retrieved from the National Ambulatory Care Reporting System [22]. Data about cancer-related consultations, decisions, and treatments, including systemic therapy and radiation therapy, were retrieved from the Activity Level Reporting database [22]. Data about approved funding requests for systemic therapy were retrieved from the New Drug Funding Program database [22]. Additional data about systemic treatment with targeted or endocrine therapy for Ontario residents age 65 and over or on social assistance were retrieved from the Ontario Drug Benefit database [22]. Due to Ontario Health (Cancer Care Ontario)’s designation as a “prescribed entity” for the purposes of Section 45 (1) of the Personal Health Information Protection Act of 2004, an ethics review was not required.

### 2.2. Index Test: Developing the Algorithm

**Figure 1:**
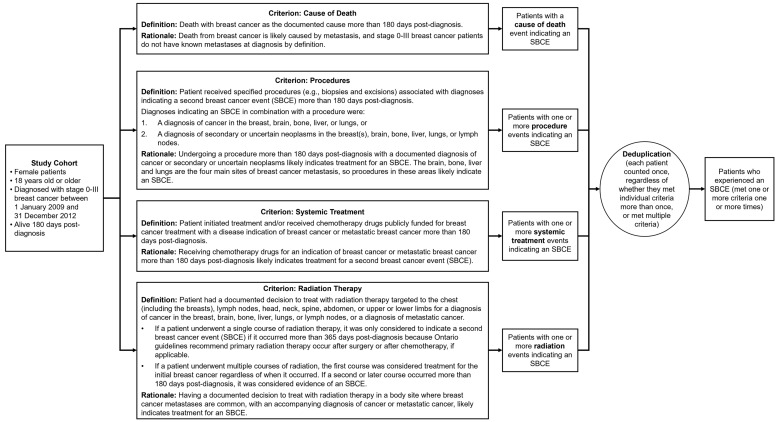
Algorithm criteria with definitions and rationale. Each criterion was applied to the entire study cohort. Patients could meet a single criterion multiple times or meet multiple criteria. For this study, we considered patients to have experienced a second breast cancer event (SBCE) if they met one criterion one time between 180 days post-diagnosis and their death or the end of follow-up.

An expert panel including surgical, medical, and radiation oncologists with expertise in breast cancer management determined algorithm criteria, i.e., types of healthcare events likely to indicate an SBCE. Criteria were based on standard-of-care curative treatments that each breast cancer patient in Ontario should be offered (Figure 1). Time frames for algorithm criteria were based on clinicians’ expertise and their review of study cohort data indicating when healthcare events for each criterion occurred relative to diagnosis. The algorithm was applied to each patient’s data starting at 180 days post-diagnosis through death or the end of the follow-up period in order to distinguish between treatment for the incident breast cancer and treatment for an SBCE. Breast cancer-related healthcare events that occurred within 180 days after the diagnosis date were considered to indicate management of the initial breast cancer, local progression, or distant disease that was occult at diagnosis.

All criteria were applied to the entire patient cohort and could be applied in any order. A patient only had to meet one of the criteria one time to be considered as having an SBCE. For the criteria based on procedures and radiotherapy treatments, probable contralateral second primary breast cancers could be identified among SBCEs in the breast based on the laterality of procedures and diagnoses. See Appendix A for code lists for each criterion.

### 2.3. Manual Record Review

A manual record review, the reference standard test, was conducted for a sub-cohort of patients seen at the Odette Cancer Center in Toronto, Canada, and the Juravinski Cancer Center in Hamilton, Canada. We calculated, a priori, the number of records required for review to accurately validate the algorithm given the prevalence of recurrence in patients with stages I, II, and III breast cancer. Stages I and II breast cancer are diagnosed much more often than stage III breast cancer, but stage III breast cancer patients are more likely to experience an SBCE [23]. To ensure sufficient statistical power (a sufficient number of patients with SBCEs in the validation sub-cohort), we sampled approximately 1000 patients with stages I, II, and III breast cancer, representing each stage at equal proportions rather than picking a random sample that would reflect the natural incidence of each stage in the population. Stage III breast cancer patients, therefore, represented a larger proportion of the validation sub-cohort than their proportion in the entire cohort. Assuming recurrence rates of 2%, 7.7%, and 20% for stage I, II, and III patients, respectively, we aimed to be able to detect an algorithm sensitivity of 75%, 85%, and 90% for stages I, II and III, and specificity of 99%, 95%, and 90% for stages I, II, and III breast cancer patients, respectively. Sampling 1000 patients of each stage (total n = 3000), we expected to observe sensitivity and specificity in the ranges of 52–91% and 98–100% for stage I; 75–92% and 93–96% for stage II; and 85–94% and 88–92% for stage III breast cancer patients. Approximately equal numbers of stage I, II, and III patients were randomly selected from each cancer center for the validation sub-cohort.

Clinical research professionals unaware of the algorithm’s SBCE classifications manually reviewed sub-cohort records. If patients met manual review criteria for experiencing an SBCE, the evidence (clinical, radiological, or tissue-based), anatomical location, and treatment information were documented. When SBCE status was unclear, the study leader at the center (A.E. or J.S.) would adjudicate. If SBCE status remained indeterminate, patients were excluded from the manual record review.

Manual review results were linked to administrative data and algorithm classifications using patients’ medical record numbers. A member of the study team (C.H.) re-reviewed administrative and manually collected data for all false-positive cases (patients classified as experiencing an SBCE by the algorithm but not reviewers). Administrative documents clearly indicative of an SBCE (e.g., a pathology report showing breast cancer or a record of systemic therapy for metastatic breast cancer) were considered more accurate than the results of a manual record review at a single center, as patients may have been diagnosed and/or treated at different centers.

### 2.4. Statistical Methods

Patient characteristics were summarized as counts with proportions for categorical data and means with standard deviations for continuous data. For continuous variables with skewed distributions, medians and interquartile ranges were used. Patients excluded during the manual record review were compared with patients who remained in the validation sub-cohort using Pearson’s chi-squared tests and a Cochran–Mantel–Haenszel statistic [24] (Appendix B). Algorithm diagnostic accuracy was assessed by calculating agreement statistics: sensitivity, specificity, positive predictive value (PPV), negative predictive value (NPV), accuracy, kappa, and prevalence-adjusted bias-adjusted kappa (PABAK), due to criticism of the kappa statistic for its dependence on outcome prevalence [25,26,27,28]. Additional agreement statistics were calculated to verify that including patients with prior cancer diagnoses did not affect algorithm diagnostic accuracy (Appendix C). Analyses were performed using SAS^®^ software version 9.4 for Microsoft Windows. Copyright © 2013 SAS Institute Inc., Cary, NC, USA.

## 3. Results

### 3.1. Cohort Characteristics and Algorithm Classifications

**Figure 2:**
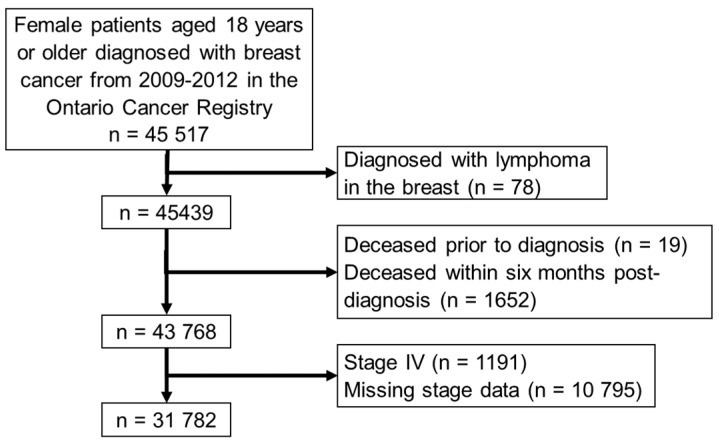
Patient inclusion/exclusion criteria.

The study cohort included 31,782 patients (Figure 2); the median follow-up time was 34 months (approximately 2.8 years; Table 1).

**Figure 3:**
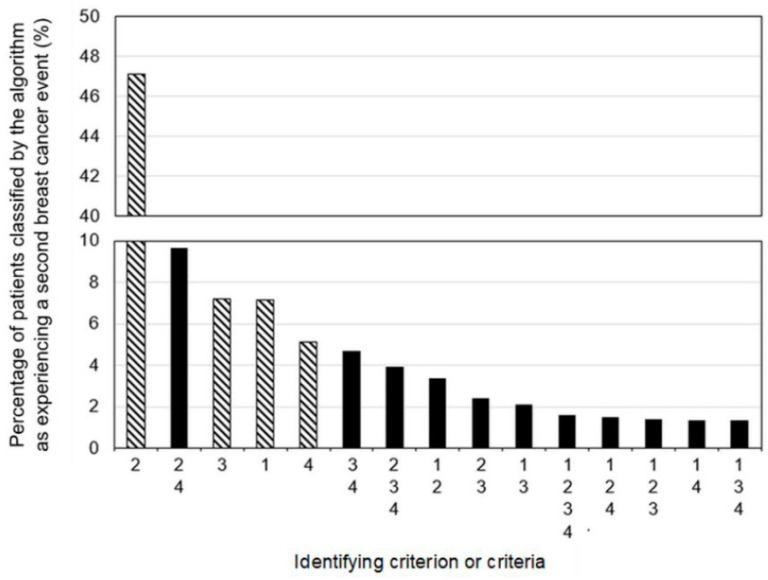
Proportions of patients classified by the algorithm as experiencing a second breast cancer event based on a single criterion (lined bars) or combinations of criteria (solid bars). Criterion/criteria groups are mutually exclusive and collectively exhaustive. All criteria were applied to the entire cohort and could be applied in any order: 1—death from breast cancer; 2—procedure and associated diagnosis; 3—systemic treatment; 4—radiotherapy.

The algorithm classified 3796 patients as experiencing an SBCE based on a maximum of 6109 events (true total unavailable due to small cell suppression of cause-of-death data by stage) for an SBCE rate of 11.9% (Table 2). Procedure and diagnosis data classified the most patients as experiencing an SBCE and events as indicating an SBCE of any criterion, followed by radiation data, systemic treatment data, and cause-of-death data (Figure 3). Notably, for all criteria except the cause of death criterion, more healthcare events indicating an SBCE were identified than patients experiencing the events, suggesting that some patients who met the criterion met it based on multiple events.

### 3.2. Exclusions during Manual Review and Validation Sub-Cohort Characteristics

Of the 3258 patients selected for the manual record review, 1013 patients were excluded because their records could not be retrieved, they did not have sufficient records for review at a study center, or their SBCE status was indeterminate. The remaining validation sub-cohort was 2245 patients (Table 3).

Pearson’s chi-squared tests indicated a potential relationship between stage at diagnosis and likelihood of exclusion during manual review based on a marginally significant *p*-value of 0.044 (Table A8). The Cochran–Mantel–Haenszel statistic [24] demonstrated that after controlling for the stage at diagnosis, more excluded patients were classified by the algorithm as having an SBCE (Table A9; *p*-value < 0.0136).

### 3.3. Algorithm Diagnostic Accuracy

After a case-by-case review of false-positive results (patients classified as experiencing an SBCE by the algorithm but not by manual review), 16 patients’ manual review SBCE statuses were revised due to definitive evidence of SBCEs in administrative data, making them true positive. Algorithm and manual review SBCE classifications after this revision are compared in Table 4A,B. The algorithm had a sensitivity of 85%, a specificity of 94%, a PPV of 67%, an NPV of 98%, a kappa of 71%, and a PABAK of 85% (Table 4C).

Prior cancer history did not observably affect the algorithm’s diagnostic accuracy, though this may be attributable to the small proportion of patients with prior cancer history (Appendix C).

## 4. Discussion

Our study demonstrates the feasibility of quantifying SBCE rates in populations by analyzing administrative data using new methods. The sensitivity and specificity of our algorithm were comparable or superior to previously published SBCE [1,2,16,19,29] and recurrence identification [3,15,17] algorithms, though the PPV was slightly lower. Our algorithm may, therefore, be useful in scenarios where the overestimation of the SBCE rate is less important (e.g., system capacity planning). High specificity and NPV make our algorithm useful for identifying patients unlikely to have experienced an SBCE (e.g., for studies about interventions to reduce recurrence rates). The overall accuracy of 92% supports our algorithm’s appropriateness for use in health system monitoring and exceeds the acceptable accuracy threshold chosen by Livaudais-Toman et al. [30].

The sensitivity of the algorithm was limited by the lack of important data in administrative databases. Some patients with SBCEs likely received treatments that were not specific to breast cancer, such as palliative care, or treatments not reported in administrative data, such as endocrine therapy in patients under age 65 and not on social assistance. Since the proportions of such patients are likely to remain constant, it may be possible to apply a correction to, or acknowledge a probable false-negative rate in, estimates of SBCE prevalence.

The relatively low PPV was attributable to false-positive SBCE classifications by the algorithm, i.e., treatments meeting criteria though they were probably not indicated for SBCEs. For example, surgical procedures occurring more than six months following a diagnosis such as a mastectomy with or without reconstruction may have reflected prophylactic treatment, patients’ aesthetic preferences, or potentially primary treatment after neoadjuvant chemotherapy. Other false positives were attributable to the limitations of manual record reviews: Some patients were erroneously determined not to have an SBCE during the manual review because they received care at multiple centers due to treatment availability or personal relocation. This likely also explains the increased rate of SBCEs according to the algorithm among patients whose records were excluded from the manual review.

Each algorithm criterion appears relevant since each criterion identified different patients. Procedure and associated diagnosis data seem especially useful, though further research is required to determine the accuracy of each criterion. Investigating why some patients were only identified posthumously based on the cause-of-death data could elucidate gaps or suggest how many patients do not receive SBCE-specific therapy.

Although we developed our algorithm from a population, a larger and more diverse group than some other authors used to develop algorithms, adjusting individual criteria or the data observation period to align with previously published algorithms could potentially improve performance. Other authors analyzed data starting after a longer time post-diagnosis or after completion of each patient’s primary treatment [1,2,3]; similar changes might reduce our false-positive rate and improve PPV. Other SBCE and breast cancer recurrence identification algorithms have incorporated different types of healthcare events [3,19], numbers [1,3] or rates of occurrence [1,2,19] of events, or intervals between events [1,2]. Promisingly, some SBCE algorithms generated by machine learning used similar criteria to those chosen by clinical experts for our algorithm [1,2].

There are some limitations to our study. Excluding patients from the validation sub-cohort during the manual record review may have led to unmeasured differences between the final sub-cohort and the entire cohort. Reviewing patient records at academic tertiary care centers offering specialized treatments may have increased the inclusion of patients who received care at multiple centers, impeding the review of comprehensive treatment records. Inter-rater reliability was not measured, though chart reviewers and study leaders met regularly to maximize consistency. Finally, we applied our algorithm to data from six months post-breast cancer diagnosis to a maximum of four years post-diagnosis, which does not represent the entire at-risk period for SBCEs. The algorithm’s accuracy may differ depending on the duration of follow-up.

## 5. Conclusions

Despite these limitations, we calculated an SBCE rate with acceptable accuracy for healthcare system monitoring by applying an algorithm to administrative data. The algorithm may be applicable to other patient populations or other cancer types with similar patterns of treatment since the data types used to identify second cancer events were not specific to breast cancer. Future developments may include adjusting algorithm criteria, incorporating additional administrative datasets, or experimenting with machine learning methods, which could potentially improve algorithm performance and expand algorithm utility.

## Figures and Tables

**Table 1:** Cohort Description.

Characteristic	Stage at Diagnosis, N (% of Stage Total)			
	Stage 0 N = 1528	Stage I N = 13,575	Stage II N = 12,141	Stage III N = 4538
Death during follow-up	6 (0.4%)	271 (2.0%)	583 (4.8%)	490 (10.8%)
Median follow-up in months (IQR)	30.3 (22.4, 40.5)	35.0 (23.4, 46.4)	34.0 (22.9, 46.4)	32.9 (21.5, 45.0)
Median age at diagnosis (IQR)	60.0 (52.0, 68.0)	63.0 (54.0, 71.0)	61.0 (50.0, 73.0)	58.0 (48.0, 71.0)
Substage at diagnosis				
0	1528 (100.0%)			
I		3552 (26.2%)		
IA		9508 (70.0%)		
IB		515 (3.8%)		
II			277 (2.3%)	
IIA			7774 (64.0%)	
IIB			4090 (33.7%)	
III				275 (6.1%)
IIIA				2538 (55.9%)
IIIB				785 (17.3%)
IIIC				898 (19.8%)
IIINOS				42 (0.9%)
Median tumor size, mm (IQR)	15.0 (7.0, 25.0)	12.0 (9.0, 16.0)	26.0 (22.0, 35.0)	45.0 (28.0, 65.0)
Patients missing tumor size data	1502 (98.3%)	4245 (31.3%)	3783 (31.2%)	1696 (37.4%)
Year of diagnosis				
2009 ^1^	17 (1.1%)	2869 (21.1%)	2776 (22.9%)	1055 (23.2%)
2010	528 (34.6%)	3620 (26.7%)	3141 (25.9%)	1210 (26.7%)
2011	506 (33.1%)	3612 (26.6%)	3117 (25.7%)	1153 (25.4%)
2012	477 (31.2%)	3474 (25.6%)	3107 (25.6%)	1120 (24.7%)
Laterality of original breast cancer diagnosis				
Right	715 (46.8%)	6925 (51.0%)	6141 (50.6%)	2292 (50.5%)
Left	818 (53.5%)	6849 (50.5%)	6193 (51.0%)	2309 (50.9%)
Tumor morphology				
Ductal carcinoma	49 (3.2%)	8069 (59.4%)	6657 (54.8%)	2296 (50.6%)
Lobular carcinoma	<6	549 (4.0%)	730 (6.0%)	313 (6.9%)
Mixed carcinoma	0	1030 (7.6%)	896 (7.4%)	315 (6.9%)
Sarcoma	0	<6	43 (0.4%)	<6
Other	<6	41–45	95 (0.8%)	19–24
Invasive cancer, missing morphology	1477 (96.7%)	3881 (28.6%)	3720 (30.6%)	1589 (35.0%)
Tumor estrogen receptor				
Borderline or positive	8 (0.5%)	8193 (60.4%)	6438 (53.0%)	2140 (47.2%)
Negative	8 (0.5%)	1041 (7.7%)	1620 (13.3%)	706 (15.6%)
Missing ^2^	1512 (99.0%)	4341 (32.0%)	4083 (33.6%)	1692 (37.3%)
Tumor progesterone receptor				
Borderline or positive	<6	7461 (55.0%)	5795 (47.7%)	1858 (40.9%)
Negative	9–13	1765 (13.0%)	2258 (18.6%)	980 (21.6%)
Missing ^2^	1514 (99.1%)	4349 (32.0%)	4088 (33.7%)	1700 (37.5%)
Tumor human epidermal growth factor receptor 2 (HER2) status				
Negative or equivocal	<6	7266 (53.5%)	6066 (50.0%)	1944 (42.8%)
Positive	<6	727 (5.4%)	997 (8.2%)	543 (12.0%)
Missing ^2^	1523 (99.7%)	5582 (41.1%)	5078 (41.8%)	2051 (45.2%)

Abbreviations: IQR, interquartile range; mm, millimeters; N, number; NOS, not otherwise specified; SBCE, second breast cancer event. ^1^ Fewer patients were diagnosed with breast cancer in 2009 because Ontario changed diagnostic criteria in 2010 to use the Surveillance, Epidemiology, and End Results system. ^2^ Biomarker status is not routinely tested in patients with ductal carcinoma in situ. Missing biomarker data for this cohort are likely due to methods of biomarker reporting to the Ontario Cancer Registry, rather than biomarker status not being measured.

**Table 2:** Algorithm classifications of second breast cancer events (SBCEs) in the entire cohort.

Characteristic	Stage at Diagnosis, N (% of Stage Total)			
	Stage 0 N = 1528	Stage I N = 13,575	Stage II N = 12,141	Stage III N = 4538
Algorithm classifications				
Patients with SBCEs	62 (4.1%)	760 (5.6%)	1635 (13.5%)	1339 (29.5%)
Patients with probable contralateral second primary breast cancers ^1^	24 (1.6%)	122 (0.9%)	146 (1.2%)	86 (1.9%)
Algorithm classifications by data type (criterion)				
Cause of death data				
Patients with SBCEs	<6	65 (0.5%)	301 (2.5%)	381 (8.4%)
Procedure and associated diagnosis data				
Patients with SBCEs	56 (3.7%)	625 (4.6%)	1158 (9.5%)	867 (19.1%)
Events	59	654	1238	961
Contralateral events	23	104	99	55
Systemic treatment data				
Patients with SBCEs	7 (0.5%)	82 (0.6%)	356 (2.9%)	486 (10.7%)
Events	7	92	402	549
Radiation therapy data				
Patients with SBCEs	12 (0.8%)	188 (1.4%)	492 (4.1%)	425 (9.4%)
Events	15	220	615	545
Contralateral events	7	50	66	45
Manual record review location				
No review	1528 (100.0%)	12,874 (94.8%)	11,329 (93.3%)	3806 (83.9%)
Juravinski Cancer Centre	0 (0%)	433 (3.2%)	474 (3.9%)	416 (9.2%)
Odette Cancer Centre	0 (0%)	268 (2.0%)	338 (2.8%)	316 (7.0%)
Death during follow-up	6 (0.4%)	271 (2.0%)	583 (4.8%)	490 (10.8%)
Median follow-up in months (IQR)	30.3 (22.4, 40.5)	35.0 (23.4, 46.4)	34.0 (22.9, 46.4)	32.9 (21.5, 45.0)
History of primary cancer before cohort entry				
Prior breast and non-breast cancer	7 (0.5%)	66 (0.5%)	37 (0.3%)	15 (0.3%)
Prior breast cancer only	84 (5.5%)	623 (4.6%)	405 (3.3%)	115 (2.5%)
Prior non-breast cancer only	76 (5.0%)	825 (6.1%)	685 (5.6%)	227 (5.0%)
No prior cancer	1361 (89.1%)	12,061 (88.8%)	11,014 (90.7%)	4181 (92.1%)

Abbreviations: IQR, interquartile range; mm, millimeters; N, number; NOS, not otherwise specified; SBCE, second breast cancer event. ^1^ Patients classified as having contralateral second primary breast cancers according to the criteria based on procedures and radiotherapy treatments are a subset of patients classified as having an SBCE.

**Table 3:** Validation sub-cohort characteristics.

Characteristic	Stage, N (%)			
	Stage I N = 701	Stage II N = 812	Stage III N = 732	Total N = 2245
Death during follow-up	14 (2.0%)	31 (3.8%)	73 (10.0%)	118 (5.3%)
Median follow-up in months (IQR)	34.8 (23.5, 47.5)	36.1 (23.5, 47.8)	31.2 (21.3, 44.4)	34.1 (22.8, 46.6)
Median age at diagnosis (IQR)	59.0 (51.0, 68.0)	58.0 (49.0, 68.0)	55.5 (47.0, 66.0)	57.0 (49.0, 67.0)
History of primary cancer before cohort entry				
Prior breast cancer (alone or with non-breast cancer)	24 (3.4%)	25 (3.0%)	14 (1.9%)	63 (2.8%)
Prior non-breast cancer	32 (4.6%)	36 (4.4%)	36 (4.9%)	104 (4.6%)
No prior cancer	645 (92.0%)	751 (92.5%)	682 (93.2%)	2078 (92.6%)
Year of diagnosis				
2009	165 (23.5%)	205 (25.2%)	167 (22.8%)	537 (23.9%)
2010	169 (24.1%)	216 (26.6%)	177 (24.2%)	562 (25.0%)
2011	187 (26.7%)	191 (23.5%)	190 (26.0%)	568 (25.3%)
2012	180 (25.7%)	200 (24.6%)	198 (27.0%)	578 (25.7%)
Substage at diagnosis				
I	201 (28.7%)			201 (9.0%)
IA	472 (67.3%)			472 (21.0%)
IB	28 (4.0%)			28 (1.2%)
II		21 (2.6%)		21 (0.9%)
IIA		490 (60.3%)		490 (21.8%)
IIB		301 (37.1%)		301 (13.4%)
III or IIINOS			34 (4.6%)	34 (1.5%)
IIIA			436 (59.6%)	436 (19.4%)
IIIB			108 (14.8%)	108 (4.8%)
IIIC			154 (21.0%)	154 (6.9%)
Median tumor size, mm (IQR)	13.0 (10.0, 17.0)	28.0 (22.0, 35.0)	52.0 (30.0, 70.0)	25.0 (15.0, 41.0)
Patients missing tumor size data	233 (33.2%)	282 (34.7%)	267 (36.5%)	782 (34.8%)
Laterality of original diagnosis				
Right	363 (51.8%)	390 (48.0%)	360 (49.2%)	1113 (49.6%)
Left	337 (48.1%)	427 (52.6%)	377 (51.5%)	1141 (50.8%)
Tumor morphology				
Ductal carcinoma	418 (59.6%)	446 (54.9%)	360 (49.2%)	1224 (54.5%)
Lobular carcinoma	22 (3.1%)	45 (5.5%)	62 (8.5%)	129 (5.7%)
Mixed carcinoma	36–40	34–38	51 (7.0%)	127 (5.7%)
Sarcoma	0	0	<6	<6
Other	<6	<6	<6	4–8
Invasive cancer, missing morphology	220 (31.4%)	282 (34.7%)	254 (34.7%)	756 (33.7%)
Tumor estrogen receptor				
Borderline or positive	403 (57.5%)	405 (49.9%)	332 (45.4%)	1140 (50.8%)
Negative	63 (9.0%)	121 (14.9%)	132 (18.0%)	316 (14.1%)
Missing ^1^	235 (33.5%)	286 (35.2%)	268 (36.6%)	789 (35.1%)
Tumor progesterone receptor				
Borderline or positive	367 (52.4%)	365 (45.0%)	286 (39.1%)	1018 (45.3%)
Negative	99 (14.1%)	161 (19.8%)	176 (24.0%)	436 (19.4%)
Missing ^1^	235 (33.5%)	286 (35.2%)	270 (36.9%)	791 (35.2%)
Tumor human epidermal growth factor receptor 2 (HER2) status				
Negative or equivocal	379 (54.1%)	407 (50.1%)	341 (46.6%)	1127 (50.2%)
Positive	43 (6.1%)	77 (9.5%)	86 (11.7%)	206 (9.2%)
Missing ^1^	279 (39.8%)	328 (40.4%)	305 (41.7%)	912 (40.6%)

Abbreviations: IQR, interquartile range; mm, millimeter; N, number; NOS, not otherwise specified; SBCE, second breast cancer event. ^1^ Missing biomarker data for this cohort is likely due to methods of biomarker reporting to the Ontario Cancer Registry, rather than biomarker status not being measured.

**Table 4:** (**A**) Algorithm and manual review classifications of second breast cancer events (SBCEs) in the validation sub-cohort; (**B**) comparison of algorithm and manual record review classifications of patients as experiencing a second breast cancer event (SBCE); (**C**) algorithm diagnostic accuracy at classifying patients as experiencing a second breast cancer event (SBCE).

(A)							
Characteristic				Stage, N (%)			
				Stage I N = 701	Stage II N = 812	Stage III N = 732	Total N = 2245
Manual review classifications ^1^							
Patients with SBCEs				27 (3.9%)	83 (10.2%)	182 (24.9%)	292 (13.0%)
Patients with probable contralateral second primary breast cancers ^2^				<6	5–10	11 (1.5%)	22 (1.0%)
Algorithm SBCE classifications							
Patients with SBCEs				48 (6.8%)	107 (13.2%)	216 (29.5%)	371 (16.5%)
Patients with likely contralateral second primary breast cancers ^2^				7 (1.0%)	11 (1.4%)	22 (3.0%)	40 (1.8%)
Algorithm classifications by data type (criterion)							
Cause of death data							
Patients				<6	28–32	68 (9.3%)	101 (4.5%)
Procedure and diagnosis data							
Patients with SBCEs				36 (5.1%)	71 (8.7%)	134 (18.3%)	241 (10.7%)
Events				37	79	159	275
Contralateral events				6	7	13	26
Systemic treatment data							
Patients with SBCEs				9 (1.3%)	42 (5.2%)	88 (12.0%)	139 (6.2%)
Events				9	42	93	144
Radiation therapy data							
Patients with SBCEs				20 (2.9%)	47 (5.8%)	89 (12.2%)	156 (6.9%)
Events				25	61	112	198
Contralateral events				<6	5–9	13	23
Manual record review location							
Juravinski Cancer Centre				433 (61.8%)	474 (58.4%)	416 (56.8%)	1323 (58.9%)
Odette Cancer Centre				268 (38.2%)	338 (41.6%)	316 (43.2%)	922 (41.1%)
**(B)**							
**Algorithm Classifications (N)**				**Manual Record Review (N)**		**Total**	
				**No SBCE**	**SBCE ^1^**		
No SBCE				1831	43	1874	
SBCE				122	249	371	
Total				1953	292	2245	
**(C)**							
**N**	**Agreement Statistic** **% (95% Confidence Interval)**						
	**Sensitivity**	**Specificity**	**Positive Predictive Value**	**Negative Predictive Value**	**Accuracy**	**Kappa ^3^**	**Prevalence-Adjusted Bias-Adjusted Kappa ^3^**
2245	85.3 (80.7–89.1)	93.8 (92.6–94.8)	67.1 (62.1–71.9)	97.7 (96.9–98.3)	92.7 (91.5–93.7)	70.9 (66.7–75.0)	85.3 (83.0–87.4)

Abbreviations: IQR, interquartile range; mm, millimeter; N, number; NOS, not otherwise specified; SBCE, second breast cancer event. ^1^ Manual review classifications in this table account for the 16 patients whose manual review SBCE status was updated from “no SBCE” to “SBCE” after case-by-case review based on definitive evidence of SBCE in administrative data. ^2^ Patients classified as having contralateral second primary breast cancers are a subset of patients classified as having an SBCE. ^3^ The Fleiss method of confidence interval calculation was used to calculate the confidence intervals for the kappa and prevalence-adjusted bias-adjusted kappa statistics [28].

## Data Availability

Data de-identified to a level suitable for public release may be provided upon request to the corresponding author, due to privacy restrictions. Ontario Health is prohibited from making the data used in this research publicly accessible if they include potentially identifiable personal health information and/or personal information as defined in Ontario law, specifically the Personal Health Information Protection Act (PHIPA) and the Freedom of Information and Protection of Privacy Act (FIPPA).

## Appendix A

### Appendix A.1. Algorithm Criteria Codes

Please note that criteria were applied to patient data from six months (180 days) after breast cancer diagnosis through the end of follow-up on 31 December 2013 or patient death, whichever came first. For the radiation therapy criterion, if a patient underwent a single course of radiation therapy, it was only considered to indicate a second breast cancer event (SBCE) if it occurred more than 365 days post-diagnosis because Ontario guidelines recommend primary radiation therapy occur after surgery or after chemotherapy, if applicable. If a patient underwent multiple courses of radiation, the first course was considered treatment for the initial breast cancer regardless of when it occurred. If a second or later course occurred more than 180 days post-diagnosis, it was considered evidence of an SBCE.

### Appendix A.2. Death from Breast Cancer Criterion

Patients met the cause of death criterion if their cause of death was coded as breast cancer, as listed below.

Data Source(s): Death records from the Ontario Registrar General.
Coding system: International Classification of Diseases, version 10 (ICD10).

**Table A1:** Death record code indicating death from a second breast cancer event.

Code(s)	Code Description
C509	Malignant neoplasm of breast, unspecified

### Appendix A.3. Procedure and Diagnosis Criterion

Patients met the procedure and diagnosis criterion if they underwent one of the procedures listed associated with one of the diagnoses listed.

#### Appendix A.3.1. Procedures

Data Source(s): Discharge Abstract Database, National Ambulatory Care Reporting System.
Coding system: Canadian Classification of Health Interventions, versions 2009, 2012, and 2015.

**Table A2:** Procedure codes for the procedure and associated diagnosis criterion.

Canadian Classification of Health Interventions Code	Canadian Classification of Health Interventions Code Description
1AA80SZXXL	Repair mening brn cranial flap OA xenogr
1AA87SZ	Excision partial, meninges and dura mater of brain using apposition technique [e.g., suture]
1AA87SZXXN	Excis prt mening brn cranial flap OA synth mat
1AC27JX	Radiation, ventricles of brain using focused beam [e.g., gamma knife, cyber knife stereotactic radiosurgery]
1AC52MBSJ	Drainage, ventricles of brain burr hole technique drainage to skin (of head) catheter or shunt (temporarily) left in situ
1AC52SE	Drainage, ventricles of brain burr hole technique drainage without shunt or catheter left in situ
1AF87DAGX	Excision partial, pituitary region endoscopic (via sinus) approach with device NEC
1AJ87SZAZ	Excision partial, cerebellum open [craniotomy flap] approach with ultrasonic aspirator [e.g., CUSA]
1AJ87SZGX	Excision partial, cerebellum open [craniotomy flap] approach with device NEC
1AN27JA	Radiation, brain using external beam [for teletherapy NEC]
1AN27JX	Radiation, brain using focused beam [e.g., gamma knife, cyber knife stereotactic radiosurgery]
1AN53SEFT	Implantation of internal device, brain burr hole technique for access of [semipermeable] catheter [e.g., for chemical palliative infusion]
1AN53SZFT	Implantation of internal device, brain craniotomy [or craniectomy] flap technique for access of [semipermeable] catheter [e.g., for chemical palliative infusion]
1AN87SEAZ	Excision partial, brain burr hole technique for access with ultrasonic aspirator [e.g., CUSA]
1AN87SZAG	Excision partial, brain craniotomy [or craniectomy] flap technique for access with laser
1AN87SZAZ	Excision partial, brain craniotomy [or craniectomy] flap technique for access with ultrasonic aspirator [e.g., CUSA]
1AN87SZGX	Excision partial, brain craniotomy [or craniectomy] flap technique for access with device NEC
1AW27JA	Radiation, spinal cord using external beam [for teletherapy NEC]
1AX35HAM0	Pharmacotherapy (local), spinal canal and meninges Percutaneous (needle) approach using antineoplastic agent NEC
1AX35HAP1	Pharmacotherapy (local), spinal canal and meninges percutaneous [needle] approach using anesthetic agent
1AX52MESJ	Drainage, spinal canal and meninges open approach shunt terminating in abdominal cavity [e.g., lumboperitoneal shunt]
1AX87LAGX	Excision partial, spinal canal and meninges using extradural incision technique [e.g., for space occupying lesion of canal] open approach with combined sources of tissue for closure with device NEC
1AX87WKGX	Excision partial, spinal canal and meninges using intradural incision technique [e.g., for meningeal mass] open approach with apposition technique [e.g., suturing] with device NEC
1EA27JA	Radiation, cranium using external beam
1EA87LANW	Excision partial, cranium open approach no tissue used [for closure of wound] using plate, screw device (with or without wire or mesh)
1EA87LANWN	Excise prt cranium OA &plate/scrw synth mater
1EA92LYXXA	Exc rad w reconstruct cranium cranial base oth appr autogr
1EQ27JA	Radiation, soft tissue of head and neck using external beam
1FM87VW	Excision partial, parotid gland using open approach with preservation of facial nerve technique
1GM59BAGX	Destruction, bronchus NEC using endoscopic per orifice approach and device NEC
1GR87DA	Excision partial, lobe of lung using endoscopic approach [VATS]
1GR87QB	Excision partial, lobe of lung using open thoracic approach
1GR89DA	Excision total, lobe of lung using endoscopic approach [VATS]
1GR89QB	Excision total, lobe of lung using open thoracic approach
1GR91QB	Excision radical, lobe of lung open thoracic approach with simple closure
1GR91QBXXN	Excise rad lobe lung thor OA synth mater
1GT27JA	Radiation, lung NEC using external beam
1GT80LA	Repair, lung NEC using open approach
1GT87DA	Excision partial, lung NEC using endoscopic approach [VATS]
1GT87QB	Excision partial, lung NEC using open thoracic approach
1GT89DA	Excise tot lung EA
1GV52DA	Drainage, pleura using endoscopic approach [VATS]
1GV52DATS	Drainage, pleura using endoscopic approach and leaving drainage tube in situ
1GV52HA	Drainage, pleura using percutaneous (needle) approach
1GV52HAHE	Drainage, pleura using percutaneous catheter (intracostal) with underwater seal drainage system
1GV52HATK	Drainage, pleura using percutaneous catheter with suction pump, (under water seal or negative pressure)
1GV52LA	Drainage, pleura using open approach
1GV52LATS	Drainage, pleura using open approach and leaving drainage tube in situ
1GV54JATS	Management of internal device, pleura of drainage tube [e.g., thoracotomy or pleural cavity drain] using external approach
1GV59DAGX	Destruction, pleura using endoscopic approach [VATS] and device NEC
1GV59DAZ9	Destruction, pleura using endoscopic approach and chemical agent NEC
1GV59HAZ9	Destruction, pleura using percutaneous instillation of agent NEC (e.g., blood, talc)
1GV87DA	Excision partial, pleura using endoscopic approach [VATS]
1GV89DA	Excision total, pleura using endoscopic approach [VATS]
1GZ31CAND	Ventilation, respiratory system NEC invasive per orifice approach by endotracheal intubation and positive pressure
1GZ31CBND	Ventilation, respiratory system NEC non-invasive approach and positive pressure ventilation (e.g., CPAP, BIPAP)
1GZ32CAMY	Oxygenation, respiratory system NEC using bulk storage manifold system
1HA87LA	Excision partial, pericardium using open approach
1MC87LA	Excision partial, lymph node(s), cervical using open approach with no tissue
1MC87LAXXE	Excise prt lymph nd neck OA loc flp
1MC89LA	Excision total, lymph node(s), cervical using open approach with no tissue
1MC91LA	Excision radical, lymph node(s), cervical without tissue radical neck dissection
1MC91VB	Excision radical, lymph node(s), cervical without tissue modified radical neck dissection
1MD27JA	Radiation, lymph node(s), axillary using external beam
1MD87LA	Excision partial, lymph node(s), axillary using open approach
1MD89LA	Excision total, lymph node(s), axillary using open approach
1MD89LAXXE	Excise tot axil lymph nd OA loc flp
1MD89LAXXG	Excise tot axil lymph nd OA ped flp
1ME87DA	Excision partial, lymph node(s), mediastinal using endoscopic approach
1ME89DA	Excision total, lymph node(s), mediastinal using endoscopic approach
1MF27JA	Radiation, lymph node(s), intrathoracic NEC using external beam
1MF87LA	Excision partial, lymph node(s), intrathoracic NEC using open approach
1MH27JA	Radiation, lymph node(s), pelvic using external beam
1MZ27JA	Radiation, lymphatic system NEC using external beam
1NF90LAXXG	Exc tot w reconstr stom OA w jejnm
1NK87RF	Excision partial, small intestine open approach enteroenterostomy anastomosis technique
1NQ57CJ	Extraction, rectum using per orifice approach and manual technique
1NQ87TF	Excision partial, rectum open abdominal [e.g., anterior] approach colostomy (or ileostomy) with closure of rectal stump [e.g., Hartmann technique] or submucous fistula
1OA27JA	Radiation liver using external beam
1OA59HAAW	Destruction, liver percutaneous approach using radiofrequency
1OA87DA	Excision partial, liver using endoscopic (laparoscopic)approach
1OA87LA	Excision partial, liver using open approach
1OA87LAAZ	Excision partial, liver using ultrasonic aspirator device (for dissection) and open approach
1OE50BANR	Dilate bile dct EPO retro &stent
1OE52GPTS	Drainage, bile ducts using percutaneous transluminal approach [e.g., transhepatic] leaving catheter (tube) in situ
1OE89UF	Excision total, bile ducts using open approach and hepaticojejunostomy technique [for anastomosis]
1OT52HATS	Drain abd cav perc app &tube NOS
1PE52HH	Drainage, renal pelvis using percutaneous approach with insertion of tube (e.g., nephrostomy, pyelostomy)
1PE59BAAG	Destruction, renal pelvis endoscopic per orifice approach Using laser (tissue ablation)
1PM52BATS	Drain bladder EPO &tube NOS
1PM87BA	Excision partial, bladder using endoscopic per orifice approach
1PV52HA	Drainage, surgically created urinary tract using percutaneous needle aspiration
1RD89DA	Excision total, ovary with fallopian tube using endoscopic [laparoscopic] approach
1RD89LA	Excise tot ovary w fallop OA
1RM89AA	Excision total, uterus and surrounding structures using combined laparoscopic and vaginal approach
1SC27JA	Radiation, spinal vertebrae using external beam
1SC74PFNW	Fixation, spinal vertebrae open posterior approach [Includes: posterolateral approach] using screw, screw with plate or rod
1SC75LLKDN	Fuse sp vert ant OA &wire/staple synth mater
1SC75PFGXN	Fuse sp vert post OA &dev NEC synth mater
1SC75PFNWA	Fuse sp vert post OA &plate/scrw autogr
1SC75PFNWN	Fuse sp vert post OA &plate/scrw synth mater
1SC75PFNWQ	Fuse sp vert post OA &plate/scrw combo tis
1SC80HABDN	Repair sp vert perc app w balloon & synth mat
1SC80HAXXN	Repair sp vert perc injct synth mater
1SC80PF	Repair, spinal vertebrae using posterior approach
1SC89LLNWA	Excise tot sp vert ant OA &plate/scrw autogr
1SC89LLNWK	Excise tot sp vert ant OA &plate/scrw homogr
1SC89LLNWN	Excise tot sp vert ant OA &plate/scrw synth mat
1SC89LLNWQ	Excise tot sp vert ant OA &plate/scrw combo tis
1SC89LNNWN	Excis tot sp vert ant w post &plate/scrw syn mat
1SC89PFGX	Excision total, spinal vertebrae posterior approach [posterolateral approach] no tissue used (device only) using device NEC
1SC89PFNWN	Excise tot sp vert post OA &plate/scrw synth mater
1SF74HANW	Fixation, sacrum and coccyx using percutaneous approach and screw, screw with plate
1SH87LAXXE	Excise prt s t back OA loc flp
1SQ27JA	Radiation, pelvis using external beam
1SQ87LAPMN	Excise prt pelvis OA &hip endoprosth synth mat
1SY80LA	Repair m chest & abd OA apposition
1SY87LA	Excision partial, muscles of the chest and abdomen using simple apposition technique [e.g., suture, staple] (for closure of surgical defect)
1SY87LAXXE	Excise prt m chest & abd OA loc flp
1SY87LAXXF	Excise prt m chest & abd non viable free flp
1SZ27JA	Radiation, soft tissue of the chest and abdomen using external beam
1SZ87LA	Excision partial, soft tissue of the chest and abdomen using open approach and apposition [suture, staple] (to close surgical defect)
1SZ87LAXXA	Excise prt s t chest & abd OA autogr
1SZ87LAXXE	Excise prt s t chest & abd OA loc flp
1SZ87LAXXG	Excise prt s t chest & abd OA ped flp
1TK74HALQ	Fixation, humerus percutaneous approach [e.g., with closed or no reduction] fixation device alone using intramedullary nail
1TK74LALQ	Fixation, humerus open approach fixation device alone using intramedullary nail
1TK74LANW	Fixation, humerus open approach fixation device alone using plate, screw
1TK80LAXXN	Repair humerus OA synth mater
1TK87LANWN	Excise prt humerus OA &plate/scrw synth mater
1TV87LA	Excision partial, radius and ulna no tissue used (for closure of defect) using no fixative device
1TZ27JA	Radiation, arm NEC using external beam
1VA74HANV	Fixation, hip joint percutaneous approach [e.g., with closed reduction or no reduction] fixation device alone using pin, nail
1VA74LALQ	Fixation, hip joint open approach fixation device alone using intramedullary nail
1VA74LALQN	Fix hip OA & intramed nail synth mater
1VA74LANV	Fixation, hip joint open approach fixation device alone using pin, nail
1VA74LANW	Fixation, hip joint open approach fixation device alone using plate, screw
1VC74HALQ	Fixation, femur percutaneous approach [e.g., with closed reduction or no reduction] fixation device alone using intramedullary nail
1VC74LALQ	Fixation, femur open approach fixation device alone using intramedullary nail
1VC74LALQN	Fix femur OA &intramed nail synth mater
1VC74LANWQ	Fix femur OA &plate/scrw combo tis
1VC80LAKDQ	Repair femur OA &fix dev NEC combo tis
1VC87LALQ	Excision partial, femur no tissue used (for closure of defect) using intramedullary nail
1VC87LANVN	Excise prt femur OA &pin/nail synth mater
1VC87LANW	Excision partial, femur with synthetic tissue [bone cement, paste] using screw, plate and screw
1VC87LAPMN	Excise prt femur OA &endoprosth synth mat
1VC91LAPNN	Excise rad femur OA &dual comp prosth synth mater
1VD87LAXXA	Excise prt m hip & thigh OA autogr
1VQ74LALQ	Fixation, tibia and fibula open approach fixation device alone using intramedullary nail
1VQ87LANWN	Excise prt tib & fib OA &plate/scrw synth mater
1VZ27JA	Radiation, leg NEC using external beam
1YA87LA	Excision partial, scalp open [excisional] approach Without tissue repair
1YK84LAXXE	Re/construct nipple OA loc flp
1YK84LAXXQ	Re/construct nipple OA combo tis
1YK87LA	Excision partial, nipple using open excisional approach
1YK87LAXXE	Excise prt nipple OA loc flp
1YK89LA	Excision total, nipple using open approach
1YK90LAXXE	Exc tot w reconstr nipple OA loc flp
1YK90LAXXQ	Exc tot w reconstr nipple OA combo tis
1YL87LA	Excision partial, lactiferous duct using open approach
1YL89LA	Excision total, lactiferous duct using open approach
1YM27JA	Radiation, breast using external beam
1YM52HA	Drainage, breast using needle aspiration
1YM52HAAV	Drainage, breast using percutaneous approach with probe
1YM52LA	Drainage, breast using incisional approach
1YM53HAEM	Implantation of internal device, breast of brachytherapy applicator using percutaneous approach
1YM53LAEM	Implantation of internal device, breast of brachytherapy applicator using open approach
1YM54HAG2	Management of internal device, breast using percutaneous (needle) approach with synthetic agent [e.g., silicone]
1YM54HAW1	Management of internal device, breast using percutaneous (needle) approach with augmentation agent [e.g., saline, soya]
1YM55LATP	Removal of device, breast without capsulectomy of tissue expander
1YM55WJPM	Removal of device, breast with capsulectomy (with or without inframammary fold repair) of breast implant [prosthesis]
1YM72LA	Release breast OA
1YM74LA	Fixation, breast using open approach
1YM78LAXXE	Repair decr sz breast loc flp
1YM78VQ	Repair by decreasing size, breast using peri areolar round block excisional technique
1YM79LAPM	Repair by increasing size, breast open approach without tissue with implantation of prosthesis
1YM79LATP	Repair by increasing size, breast open approach without tissue with implantation of tissue expander
1YM79LATPG	Augment breast OA w tiss expandr &ped flp
1YM80LA	Repair, breast open approach without tissue with no implantation of device
1YM80LAPM	Repair, breast open approach without tissue with implantation of breast prosthesis
1YM80LAPMA	Repair breast w prosth autogr
1YM80LAPMF	Repair breast OA w prosth free flp
1YM80LAPMG	**2009**: Repair, breast using distant pedicled flap (1) with implantation of breast prosthesis **2012**: Repair, breast open approach using distant pedicled flap with implantation of breast prosthesis
1YM80LATP	Repair, breast open approach without tissue with implantation of tissue expander
1YM80LATPE	Repair breast w tiss expandr loc flp
1YM80LATPG	**2009**: Repair, breast using distant pedicled flap (1) with implantation of tissue expander **2012**: Repair, breast open approach using distant pedicled flap with implantation of tissue expander
1YM80LATPK	Repair breast OA w tiss expandr homogr
1YM80LAXXA	**2009**: Repair, breast using autograft with no implantation of device **2012**: Repair, breast open approach using autograft with no implantation of device
1YM80LAXXE	Repair breast w loc flp
1YM80LAXXF	**2009**: Repair, breast using free flap with no implantation of device **2012**: Repair, breast open approach using free flap with no implantation of device
1YM80LAXXG	**2009**: Repair, breast using distant pedicled flap with no implantation of device **2012**: Repair, breast open approach using distant pedicled flap with no implantation of device
1YM87DA	Excision partial, breast using endoscopic approach with simple apposition
1YM87GB	Excision partial, breast using endoscopic guide wire (or needle hook) excision technique with simple apposition of tissue
1YM87LA	Excision partial, breast using open approach with simple apposition of tissue (e.g., suturing)
1YM87LAXXA	Excise prt breast OA autogr
1YM87LAXXE	Excise prt breast OA loc flp
1YM87UT	Excision partial, breast using open guide wire (or needle hook) excision technique and simple apposition of tissue
1YM88LAPM	Excision partial with reconstruction, breast without tissue with implantation of prosthesis
1YM88LAPME	Exc prt breast w prosth loc flp reconst
1YM88LAPMF	Exc prt breast w prosth free flp reconstr
1YM88LAPMG	Exc prt breast w prosth ped flp reconstr
1YM88LAQF	Exc prt breast w prosth/tis expand reconstr
1YM88LAQFE	Exc prt breast w prosth/tis expand loc flp reconst
1YM88LATP	Excision partial with reconstruction, breast without tissue with implantation of tissue expander
1YM88LATPE	Exc prt breast w tiss expandr &loc flp reconst
1YM88LATPF	Exc prt breast w tiss expand free flp reconstr
1YM88LATPG	Exc prt breast w tiss expand ped flp reconstr
1YM88LATPK	Exc prt breast w tiss expand homogr reconstr
1YM88LAXXE	Exc prt breast w loc flp reconstr
1YM88LAXXF	Exc prt breast w free flp reconstr
1YM88LAXXG	Exc prt breast w ped flp reconstr
1YM89LA	Excision total, breast using open approach
1YM89LAXXA	Excise tot breast w autogr
1YM89LAXXE	Excise tot breast OA loc flp
1YM90LAPM	Excision total with reconstruction, breast simple mastectomy with no node dissection without tissue with implantation of breast prosthesis
1YM90LAPME	Exc tot breast prosth loc flp reconstr
1YM90LAPMF	Exc tot breast prosthesis free flp reconstr
1YM90LAPMG	Exc tot breast prosth ped flp reconstr
1YM90LAQF	Exc tot breast prosth w tiss expand reconstr
1YM90LAQFE	Exc tot breast prosth tis expand loc flp reconst
1YM90LAQFG	Exc tot breast prosth tis expand ped flp reconst
1YM90LATP	Excision total with reconstruction, breast simple mastectomy with no node dissection without tissue with implantation of tissue expander
1YM90LATPF	Exc tot breast tiss expand free flp reconstr
1YM90LATPG	Exc tot breast tiss expand ped flp reconstr
1YM90LAXXF	Exc tot breast free flp reconstr
1YM90LAXXG	Exc tot breast ped flp reconstr
1YM90LAXXQ	Exc tot w reconstr breast OA combo tis
1YM91LA	Excision radical, breast without tissue modified or NOS
1YM91LATP	Excision radical, breast with implantation of tissue expander modified or NOS
1YM91LAXXA	**2009**: Excision radical (modified), breast using autograft **2012**: Excision radical, breast using autograft modified or NOS
1YM91LAXXE	**2009**: Excision (modified) radical, breast using local flap **2012**: Excision radical, breast using local flap modified or NOS
1YM91TR	Excision radical, breast without tissue extended [Urban]
1YM91TRXXE	**2009**: Excision extended radical, breast using local flap **2012**: Excision radical, breast using local flap extended [Urban]
1YM92LAPME	Mod rad mastectmy w prosth loc flp reconst
1YM92LAPMF	Mod rad mastectmy w prosth free flp reconst
1YM92LAPMG	Mod rad mastectmy w prosth ped flp reconst
1YM92LAQFE	Mod rad mastectmy w prosth tiss expand loc flp
1YM92LAQFG	Mod rad mastectmy w prosth tiss expand ped flp
1YM92LATPE	Mod rad mastectmy w tiss expandr loc flp reconst
1YM92LATPF	Mod rad mastectmy w tiss expand free flp reconst
1YM92LATPG	Mod rad mastectmy w tiss expand ped flp reconst
1YM92LAXXF	Mod rad mastectmy w free flp reconst
1YM92LAXXG	Mod rad mastectmy w ped flp reconst
1YM92LAXXQ	**2009**: Excision radical with reconstruction, breast modified or NOS with no implanted device using combined sources of tissue (e.g., free and pedicled TRAM flap) **2012**: Excision radical with reconstruction, breast modified or NOS using combined sources of tissue (e.g., free and pedicled TRAM flap) with no implanted device
1YM92TRPME	Ext rad mastectmy w prosth loc flp reconst
1YM92TRTPE	Ext rad mastectmy wtiss expand loc flp reconst
1YM92TRXXQ	Exc rad w reconstr breast OA w ext rad excisn combo tis
1YR87LA	Excision partial, skin of axillary region open [excisional] approach with apposition technique (e.g., suture, glue) for closure
1YR87LAXXB	Excise prt sk axilla &splt gr
1YS87LA	Excision partial, skin of abdomen and trunk open [excisional] approach with apposition technique (suture, glue) for closure
1YS87LAXXE	Excise prt sk abd & trunk &loc flp
1ZZ35CAM0	Pharmacotherapy, total body antineoplastic and immunomodulating agents per orifice (oral) approach antineoplastic agent NOS
1ZZ35CAM2	Pharmacotherapy, total body antineoplastic and immunomodulating agents per orifice (oral) approach antimetabolite
1ZZ35CAM4	Pharmacotherapy, total body antineoplastic and immunomodulating agents per orifice (oral) approach cytotoxic antibiotic and related substance
1ZZ35CAM5	Pharmacotherapy, total body antineoplastic and immunomodulating agents per orifice (oral) approach other antineoplastic
1ZZ35HAK7	Pharm tx NEC perc app &macrolide/lincosamide
1ZZ35HAM0	Pharmacotherapy, total body antineoplastic and immunomodulating agents percutaneous needle approach [intramuscular, intravenous, subcutaneous, intradermal] antineoplastic agent NOS
1ZZ35HAM3	Pharmacotherapy, total body antineoplastic and immunomodulating agents percutaneous approach [intramuscular, intravenous, subcutaneous, intradermal] plant alkaloid and other natural product
1ZZ35HAM4	Pharmacotherapy, total body antineoplastic and immunomodulating agents percutaneous approach [intramuscular, intravenous, subcutaneous, intradermal] cytotoxic antibiotic and related substance
1ZZ35HAM5	Pharmacotherapy, total body antineoplastic and immunomodulating agents percutaneous approach [intramuscular, intravenous, subcutaneous, intradermal] other antineoplastic
1ZZ35HAM9	Pharmacotherapy, total body antineoplastic and immunomodulating agents percutaneous approach [intramuscular, intravenous, subcutaneous, intradermal] Combination [multiple] antineoplastic agents
1ZZ35HAN5	Pharmacotherapy, total body musculoskeletal system agents percutaneous approach [intramuscular, intravenous, subcutaneous, intradermal] drug for treatment of bone disease
2AX13HA	Specimen collection (diagnostic), spinal canal and meninges using percutaneous (needle) approach
2EQ71HA	Biopsy s t head & neck perc ndle app
2FU71HA	Biopsy thyr gl perc ndle app
2GM71BA	Biopsy, bronchus using endoscopic per orifice approach
2GM71BP	Biopsy, bronchus using endoscopic per orifice approach with needle aspiration
2GM71BR	Biopsy, bronchus using endoscopic per orifice approach with brushing/washing
2GT71BA	Biopsy, lung using endoscopic per orifice approach
2GT71BP	Biopsy, lung using endoscopic per orifice approach and needle aspiration
2GT71HA	Biopsy, lung using percutaneous (needle) approach
2GW71DA	Biopsy mediast endo app
2HZ24JAXJ	ECG NOS (ext applic record electrode)
2ME71BP	Biopsy, mediastinal lymph nodes endoscopic per orifice, with needle aspiration
2ME71DA	Biopsy, mediastinal lymph nodes using endoscopic approach
2ME71LA	Biopsy, mediastinal lymph nodes using open approach
2MZ71HA	Biopsy lymph sys perc ndle app
2NF71BA	Biopsy stomach EPO app
2NK70BABL	Inspect sm intest EPO app & gastroscope
2OT71DA	Biopsy, abdominal cavity using endoscopic [laparoscopic] approach
2SZ71HA	Biopsy s t chest & abd perc ndle app
2WY71HA	Biopsy bone marrow perc ndle app
2YK71HA	Biopsy, nipple using percutaneous approach (needle, punch)
2YK71LA	Biopsy, nipple using open [incisional] approach
2YM70LA	Inspection, breast NOS using open approach
2YM71HA	Biopsy, breast NOS using percutaneous (needle) aspiration
2YM71HAGX	Biopsy, breast NOS percutaneous approach using device NEC
2YM71LA	Biopsy, breast NOS incisional biopsy
2ZZ02ZX	Assessment (examination), total body for determining candidacy for treatment
2ZZ13RA	Specimen collect NEC vn puncture
3AN40WE	MRI brain with & without enhancement
3ER20WC	CT head with enhancement
3OG10WZ	Xray b dct w pancr w endo retrograde injct contr
3OT30DA	U/S abd cav alone
3SC40WE	MRI sp vert with & without enhancement
3WZ70CC	Nuclear study msk sys SPECT tomo
3YM30DA	U/S breast u/s only
7SC08PL	Ministrate NEC personal care chronic pain

#### Appendix A.3.2. Diagnoses

Data Source(s): Discharge Abstract Database, National Ambulatory Care Reporting System.
Coding system: International Classification of Diseases, version 10 (ICD10), 2015.

**Table A3:** International Classification of Diseases version 10 diagnosis codes associated with procedures that indicated a second breast cancer event.

International Classification of Diseases (Version 10) Codes	International Classification of Diseases (Version 10) Code Descriptions
C50	Malignant neoplasm of breast
C22	Malignant neoplasm of liver and intrahepatic bile ducts (excluding biliary tract NOS, secondary malignant neoplasm of liver)
C34	Malignant neoplasm of bronchus and lung
C41	Malignant neoplasm of bone and articular cartilage of other and unspecified sites
D43	Neoplasm of uncertain or unknown behaviour of brain and central nervous system (excluding peripheral nerves and autonomic nervous system)
C71	Malignant neoplasm of brain (excluding cranial nerves, retrobulbar tissue)
C77	Secondary and unspecified malignant neoplasm of lymph nodes (excluding malignant neoplasm of lymph nodes, specified as primary)
C78	Secondary malignant neoplasm of respiratory and digestive organs
C78.0	Secondary malignant neoplasm of lung
C78.3	Secondary malignant neoplasm of other and unspecified respiratory organs
C78.7	Secondary malignant neoplasm of liver and intrahepatic bile duct
D48	Neoplasm of uncertain or unknown behaviour of other and unspecified sites (excluding neurofibromatosis (nonmalignant))
D48.0	Bone and articular cartilage (excluding articular cartilage and cartilage of the ear, larynx, and nose; the connective tissue of the eyelid; and synovia).
D48.6	Breast (including connective tissue of breast, cystosarcoma phyllodes; excluding skin of breast)
D37	Neoplasm of uncertain or unknown behaviour of oral cavity and digestive organs
D37.6	Liver, gallbladder and bile ducts
D38	Neoplasm of uncertain or unknown behaviour of middle ear and respiratory and intrathoracic organs (excluding heart)
D38.1	Trachea, bronchus and lung
C79	Secondary malignant neoplasm of other and unspecified sites
C79.3	Secondary malignant neoplasm of brain and cerebral meninges
C79.4	Secondary malignant neoplasm of other and unspecified parts of nervous system
C79.5	Secondary malignant neoplasm of bone and bone marrow

### Appendix A.4. Systemic Therapy Criterion

Patients met the systemic therapy criterion if they received one of the drugs listed, in some cases, for one of the indications listed.

Data Source(s): Activity Level Reporting database.
Coding system: Not applicable.

**Table A4:** Systemic therapy data types and descriptions that indicated a second breast cancer event.

Data Type Analyzed by Algorithm	Description
Drug description	PAMIDRONATE
	CLODRONATE
	VINORELBINE
	PACLITAXEL
	ERIBULIN
	PERTUZUMAB
	TRASTUZUMAB EMTANSINE

Data Source(s): New Drug Funding Program database.
Coding system: Proprietary to Ontario Health.

**Table A5:** Disease indications and funding policy name or name of drug received by patient that indicated a second breast cancer event.

Disease Indication	Policy Name/Drug Name
Metastatic or Incurable Locally Advanced—Breast Cancer	Eribulin
Unresectable Locally Recurrent or Metastatic—Breast Cancer	Pertuzumab with Trastuzumab
	Trastuzumab Emtansine
Unresectable Locally Advanced or Metastatic Breast Cancer as Third or Subsequent Line of Treatment (Time-Limited)	Trastuzumab Emtansine
Metastatic Breast Cancer	Clodronate (IV)
	Docetaxel
	Nab-Paclitaxel
	Paclitaxel
	Pamidronate
	Trastuzumab in combination with Docetaxel
	Trastuzumab in combination with Paclitaxel
	Trastuzumab in combination with Vinorelbine
	Trastuzumab with First Line Docetaxel
	Trastuzumab—Single Agent
	Vinorelbine
Second Line—Metastatic Breast Cancer	Trastuzumab

### Appendix A.5. Radiation Treatment Criterion

Patients met this criterion if they received radiation therapy in one of the anatomical sites listed to treat one of the associated diagnoses listed in the appropriate time period.

### Appendix A.6. Body Regions Where Radiation Was Applied

Data Source(s): Activity Level Reporting database.
Coding system: Proprietary to Ontario Health.

**Table A6:** Body regions and codes for receiving radiation that indicated a second breast cancer event.

Body Region Group	Body Region Code	Body Region Code Description
ABDOMEN	ABDL	Left abdomen
ABDOMEN (continued)	ABDO	Whole abdomen
	ABDR	Right abdomen
	ABLB	Lower abdomen
	ABLL	Left lower abdomen
	ABLR	Right lower abdomen
	ABUB	Upper abdomen
	ABUL	Left upper abdomen
	ABUR	Right upper abdomen
	ADRL	Left adrenal
	ADRR	Right adrenal
	BILE	Bile duct
	COLN	Colon
	EPIG	Epigastrium
	GALL	Gall bladder
	INVY	Inverted ‘y’ (dog-leg, hockey-stick)
	KIDL	Left kidney
	KIDR	Right kidney
	LIVR	Liver
	PANC	Pancreas
	PARA	Para-aortic nodes
	SPLE	Spleen
	STOM	Stomach
CHEST	AXIL	Left axilla
	AXIR	Right axilla
	BREB	Bilateral breast
	BREL	Left breast
	BRER	Right breast
	CHEB	Bilateral chest lung & area involve
	CHEL	Left chest
	CHER	Right chest
	CHWB	Bilateral chest wall (w/o breast)
	CHWL	Left chest wall
	CHWR	Right chest wall
	CLAB	Bilateral clavicle
	CLAL	Left clavicle
	CLAR	Right clavicle
	ESOI	Lower esophagus
	ESOM	Middle esophagus
	ESOS	Upper esophagus
	ESOW	Entire esophagus
	HEML	Left hemimantle
	HEMR	Right hemimantle
	HERT	Heart
CHEST (continued)	IMCB	Bilateral internal mammary chain
	LUNB	Bilateral lung
	LUNL	Left lung
	LUNR	Right lung
	MANT	Mantle
	MEDI	Mediastinum
	PLEL	Left pleura (as in mesothelioma)
	PLER	Right pleura
	RIBL	Left ribs
	RIBR	Right ribs
	SCAB	Bilateral scapula
	SCAL	Left scapula
	SCAR	Right scapula
	SCNB	Bilateral supraclavicular nodes
	SCNL	Left supraclavicular nodes
	SCNR	Right supraclavicular nodes
	STER	Sternum
HEAD	ANTB	Bilateral antrum (bull’s eye)
	ANTL	Left antrum
	ANTR	Right antrum
	BRAI	Brain
	CHKL	Left cheek
	CHKR	Right cheek
	EARL	Left ear
	EARR	Right ear
	ETHM	Ethmoid sinus
	EYEB	Bilateral eyes
	EYEL	Left eye
	EYER	Right eye
	FACB	Bilateral face
	FACL	Left face
	FACR	Right face
	FLOO	Floor of mouth (boosts)
	FOSS	Posterior fossa
	GING	Gingiva
	HEAD	Head
	LACB	Bilateral lacrimal gland
	LACL	Left lacrimal gland
	LACR	Right lacrimal gland
	LIPB	Both lip(s)
HEAD (continued)	LIPI	Lower lip
	LIPS	Upper lip
	MANB	Bilateral mandible
	MANL	Left mandible
	MANR	Right mandible
	MAXB	Bilateral maxilla
	MAXL	Left maxilla
	MAXR	Right maxilla
	NASA	Nasal fossa
	NASO	Nasopharynx
	ORAL	Oral cavity/buccal mucosa
	ORBB	Bilateral orbit
	ORBL	Left orbit
	ORBR	Right orbit
	OROP	Oropharynx
	PALH	Hard palate
	PALS	Soft palate
	PALX	Palate unspecified
	PARL	Left parotid
	PARR	Right parotid
	PITU	Pituitary
	SALL	Left salivary gland
	SALR	Right salivary gland
	SKUL	Skull
	SPHE	Sphenoid sinus
	SUBM	Submandibular glands
	TONG	Tongue
	TONS	Tonsil
	UVUL	Uvula
LOWER LIMB	ANKB	Bilateral ankle
	ANKL	Left ankle
	ANKR	Right ankle
	FEMB	Bilateral femur
	FEML	Left femur
	FEMR	Right femur
	FIBL	Left fibula
	FIBR	Right fibula
LOWER LIMB (continued)	FOOB	Bilateral feet
	FOOL	Left foot
	FOOR	Right foot
	HEEB	Bilateral heel
	HEEL	Left heel
	HEER	Right heel
	HIPB	Bilateral hip
	HIPL	Left hip
	HIPR	Right hip
	KNEB	Bilateral knee
	KNEL	Left knee
	KNER	Right knee
	LEGB	Bilateral leg
	LEGL	Left leg
	LEGR	Right leg
	LELB	Lower bilateral leg
	LELL	Lower left leg
	LELR	Lower right leg
	LEUB	Upper bilateral leg
	LEUL	Upper left leg
	LEUR	Upper right leg
	TIBL	Left tibia
	TIBR	Right tibia
	TOEL	Left toes
	TOER	Right toes
NECK	HYPO	Hypopharynx
	LARP	Larygopharynx
	LARY	Larynx
	NECB	Bilateral neck includes nodes
	NECL	Left neck includes nodes
	NECR	Right neck includes nodes
	PYRI	Pyriform fossa (sinuses)
	THYB	Thyroid
	TRAC	Trachea
SPINE	COCC	Coccyx
	SACR	Sacrum
	SPCT	Cervical & thoracic spine
	SPIC	Cervical spine
	SPIL	Lumbar spine
	SPIT	Thoracic spine
	SPIW	Whole spine
	SPLS	Lumbo-sacral spine
	SPTL	Thoracic & lumbar spine
UPPER LIMB	ARLL	Lower left arm
	ARLR	Lower right arm
	ARMB	Bilateral arms
	ARML	Left arm
	ARMR	Right arm
	ARUL	Upper left arm
	ARUR	Upper right arm
	FING	Finger (including thumbs)
	HANB	Bilateral hand
	HANL	Left hand
	HANR	Right hand
	HUML	Left humerus
	HUMR	Right humerus
	RADL	Left radius
	RADR	Right radius
	SHOB	Bilateral shoulder
	SHOL	Left shoulder
	SHOR	Right shoulder
	ULNL	Left ulna
	ULNR	Right ulna

### Appendix A.7. Diagnoses Associated with Radiation

Data Source(s): Activity Level Reporting database.
Coding system: International Classification of Diseases, version 10 (ICD10), 2015.

**Table A7:** International Classification of Diseases version 10 diagnosis codes that indicated a second breast cancer event.

Codes	Code Description (ICD-10 Version 2015)
C50	Malignant neoplasm of breast
C34	Malignant neoplasm of bronchus and lung
C40	Malignant neoplasm of bone and articular cartilage of limbs
C71	Malignant neoplasm of brain
C77	Secondary and unspecified malignant neoplasm of lymph nodes
C78	Secondary malignant neoplasm of respiratory and digestive organs
C79	Secondary malignant neoplasm of other and unspecified sites

## Appendix B

### Exclusions during Manual Record Review and Comparison to Final Validation Sub-Cohort

Of the 3258 patients selected for manual record review, 1013 patients were excluded because their records could not be retrieved, they did not have sufficient records for review at a study center, or their SBCE status was indeterminate. The remaining validation sub-cohort was 2245 patients (main text Table 3). We conducted additional statistical analyses to determine whether patients excluded during manual record review differed from patients who remained in the validation sub-cohort. Pearson’s Chi-squared tests were used to determine whether patients excluded during manual record review differed from the patients remaining in the validation sub-cohort based on stage at diagnosis and algorithm classification as having or not having an SBCE. A Cochran-Mantel-Haenszel statistic [24] was used to test for conditional independence between remaining in the validation sub-cohort and algorithm SBCE classification after controlling for stage at diagnosis.

Pearson’s chi-squared tests indicated a potential relationship between stage at diagnosis and likelihood of exclusion during manual review based on a marginally significant *p*-value of 0.044 (main text Table 4A). The Cochran-Mantel-Haenszel statistic [24] demonstrated that after controlling for stage at diagnosis, the algorithm classified more excluded patients as having an SBCE (main text Table 4B; *p*-value < 0.0136).

**Table A8:** Stage at diagnosis among patients excluded during manual review and patients remaining in the validation sub-cohort.

Patient Group	Stage at Diagnosis			
	Stage 1 N (%)	Stage 2 N (%)	Stage 3 N (%)	Total N
Remaining validation sub-cohort	701 (31.2%)	812 (36.2%)	732 (32.6%)	2245
Excluded during manual review	347 (34.3%)	322 (31.8%)	344 (34.0%)	1013

Abbreviations: N, number.

**Table A9:** Algorithm classification as experiencing a second breast cancer event (SBCE) stratified by stage at diagnosis and exclusion during manual review.

Stage at Diagnosis	Patient Group	Algorithm SBCE Classification	
		SBCE N (Row%)	No SBCE N (Row%)
Stage 1	Remaining validation sub-cohort	48 (6.8%)	653 (93.2%)
	Excluded during manual review	27 (7.8%)	320 (92.2%)
Stage 2	Remaining validation sub-cohort	107 (13.2%)	705 (86.8%)
	Excluded during manual review	61 (18.9%)	261 (81.1%)
Stage 3	Remaining validation sub-cohort	216 (29.5%)	516 (70.5%)
	Excluded during manual review	114 (33.1%)	230 (66.9%)

Abbreviations: N, number; SBCE, second breast cancer event.

## Appendix C

### Algorithm Diagnostic Accuracy by Prior Cancer History

Algorithm diagnostic accuracy was assessed for patients with a history of cancer prior to the breast cancer diagnosis that qualified them for inclusion in this study. Diagnostic accuracy was similar for the entire cohort, patients with no prior cancer, and patients with no prior breast cancer, though sensitivity decreased for patients with any prior cancer (prior breast or non-breast cancer, or both). Patients with prior breast cancers constituted too small a group to analyze separately. The comparable diagnostic accuracy for patients with no prior cancer and no prior breast cancer suggests that inclusion of patients with prior non-breast cancers did not meaningfully affect algorithm performance.

**Table A10:** Algorithm diagnostic accuracy at classifying patients as experiencing a second breast cancer event (SBCE), stratified by prior cancer status.

Patients’ Cancer Status Prior to Cohort Entry	N	Agreement Statistic % (95% Confidence Interval)						
		Sensitivity	Specificity	Positive Predictive Value	Negative Predictive Value	Accuracy	Kappa ^1^	Prevalence-Adjusted Bias-Adjusted Kappa ^1^
Remaining validation sub-cohort	2245	85.3 (80.7–89.1)	93.8 (92.6–94.8)	67.1 (62.1–71.9)	97.7 (96.9–98.3)	92.7 (91.5–93.7)	70.9 (66.7–75.0)	85.3 (83.0–87.4)
No prior breast cancer (no prior cancer and prior non-breast cancer)	2182	85.9 (81.3–89.8)	93.7 (92.5–94.8)	66.5 (61.3–71.4)	97.9 (97.1–98.5)	92.7 (91.5–93.8)	70.8 (66.5–75.0)	85.4 (83.1–87.5)
Any prior cancer (prior breast cancer, non-breast cancer, or both)	167	79.3 (60.3–92.0)	93.5 (88.0–97.0)	71.9 (53.3–86.3)	95.6 (90.6–98.4)	91.0 (85.6–94.9)	69.9 (55.7–84.1)	82.0 (71.2–89.8)
No prior cancer	2078	85.9 (81.1–89.9)	93.8 (92.6–94.8)	66.7 (61.4–71.7)	97.9 (97.1–98.5)	92.8 (91.6–93.9)	70.9 (66.6–75.3)	85.6 (83.2–87.7)

Abbreviations: N, number; SBCE, second breast cancer event. ^1^ The Fleiss method of confidence interval calculation was used to calculate the confidence intervals for the kappa and prevalence-adjusted bias-adjusted kappa statistics [28].
